# Investigation of Damage Evolution in Heterogeneous Rock Based on the Grain-Based Finite-Discrete Element Model

**DOI:** 10.3390/ma14143969

**Published:** 2021-07-15

**Authors:** Shirui Zhang, Shili Qiu, Pengfei Kou, Shaojun Li, Ping Li, Siquan Yan

**Affiliations:** 1 State Key Laboratory of Geomechanics and Geotechnical Engineering, Institute of Rock and Soil Mechanics, Chinese Academy of Sciences, Wuhan 430071, China; zhangshirui19@mails.ucas.ac.cn (S.Z.); sjli@whrsm.ac.cn (S.L.); 2 University of Chinese Academy of Sciences, Beijing 100049, China; 3 No.2 Mining Area, Jinchuan Group Co., Ltd., Jinchang 737100, China; rouzhen1@sina.com; 4 College of Resources and Environmental Science, South-Central University for Nationalities, Wuhan 430074, China; liping@mail.scuec.edu.cn; 5 Yellow River Engineering Consulting Co., Ltd., Zhengzhou 450000, China; ysq801209@126.com

**Keywords:** combined finite-discrete element method, grain-based model, Beishan granite, grain scale effect, grain orientation effect

## Abstract

Granite exhibits obvious meso-geometric heterogeneity. To study the influence of grain size and preferred grain orientation on the damage evolution and mechanical properties of granite, as well as to reveal the inner link between grain size‚ preferred orientation, uniaxial tensile strength (UTS) and damage evolution, a series of Brazilian splitting tests were carried out based on the combined finite-discrete element method (FDEM), grain-based model (GBM) and inverse Monte Carlo (IMC) algorithm. The main conclusions are as follows: (1) Mineral grain significantly influences the crack propagation paths, and the GBM can capture the location of fracture section more accurately than the conventional model. (2) Shear cracks occur near the loading area, while tensile and tensile-shear mixed cracks occur far from the loading area. The applied stress must overcome the tensile strength of the grain interface contacts. (3) The UTS and the ratio of the number of intergrain tensile cracks to the number of intragrain tensile cracks are negatively related to the grain size. (4) With the increase of the preferred grain orientation, the UTS presents a “V-shaped” characteristic distribution. (5) During the whole process of splitting simulation, shear microcracks play the dominant role in energy release; particularly, they occur in later stage. This novel framework, which can reveal the control mechanism of brittle rock heterogeneity on continuous-discontinuous trans-scale fracture process and microscopic rock behaviour, provides an effective technology and numerical analysis method for characterizing rock meso-structure. Accordingly, the research results can provide a useful reference for the prediction of heterogeneous rock mechanical properties and the stability control of engineering rock masses.

## 1. Introduction

Natural granite is characterized by low permeability, good thermal conductivity, high strength and little deformation. Therefore, granite is often used to create a good engineering environment for tunnels, powerhouses or underground nuclear waste repositories, such as the Bayu tunnel [1], Shuangjiangkou hydropower station [2], Beishan high-level radioactive nuclear waste repository [3] and Mine-by URL [4]. Research on the mechanical properties of granite and the propagation characteristics of microcracks therein is very important for its engineering application. For instance, quantitative petrographic analysis showed that crystalline rocks exhibit mineral aggregation at the grain scale, leading to complex internal microstructures [5]. Accordingly, a thorough understanding of the effect of the meso-structure on the mechanism responsible for the initiation, propagation and coalescence of microcracks will facilitate research on the mesoscopic failure behavior of granite.

Grain-scale heterogeneity is a combination of several types of heterogeneity, including geometric heterogeneity resulting from grain shape, grain orientation and grain size; material heterogeneity resulting from the mismatch of different grains; and contact heterogeneity resulting from grain boundary anisotropy. Among them, the grain size and grain orientation, as intrinsic properties that control the heterogeneity of rock, have attracted the attention of many scholars, and many experimental and numerical studies have been carried out. Through experiments on granite and marble, Brace et al. [6] found that fine-grained rocks display a high compressive strength. Onodera et al. [7] proposed a linear relationship between the grain size and strength of igneous rock; that is, the uniaxial compressive strength (UCS) increased as the grain size decreased. Ghazvinian et al. [8] conducted experiments on the anisotropic mechanical behavior of granite and limestone, and reported that the strength and failure mode changed with the preferred grain orientation. In addition to experimental research, numerical methods, such as particle flow code (PFC) [9], universal discrete element code (UDEC) [10] and the combined finite-discrete element method [11] (FDEM) have also been used to evaluate the influence of grain size on rock strength. Wong et al. [12] proposed a method to reduce the strength parameters of grain boundaries to simulate the mechanism of the grain size effect based on PFC. Using the FDEM-GBM, Li et al. [13] found that the effect of grain size on the UCS was consistent with the findings of experiments. In fact, recrystallized quartz, as the primary mineral in granite, has the possibility to have an obviously preferred orientation in some cases. Pan et al. [14] utilized an improved UDEC-GBM and discovered that with the increase of preferred grain orientation, the UCS showed a “U-shaped” characteristic distribution.

Because the uniaxial tensile strength (UTS) determined by the Brazilian indirect tensile test of brittle rocks is much lower than UCS, brittle rocks are much more sensitive to tensile loads than to compressive loads. Thus, it is necessary to study the effect of the grain size and grain orientation on the tensile strength and the evolution of fractures. As a complement to laboratory testing, numerical simulation is also a feasible method. Therefore, this paper used finite-discrete code [15] based on the FDEM to establish a meso-scale numerical model of Beishan granite [16], and analyzed the influence of the grain size and preferred grain orientation on the UTS and damage evolution process.

This framework enables the intergranular and transgranular contacts to be modeled explicitly, while taking the actual grain morphology into consideration. Furthermore, the mesoscopic contacts of the FDEM-GBM include intergrain contacts (including homophase grain contacts and heterophase grain contacts) and intragrain contacts. Hence, this explicit modeling approach allows grain contacts to be assigned different mechanical properties. This paper is structured to initially introduce the meso-structure model, followed by a validation of the simulation results against published experimental results [16]. The FDEM-GBM can provide an efficient way to simulate grain breakage and insights into the propagation of grain-scale microcracks, which can elucidate the relationship between the evolution of the UTS and the failure mechanisms of crystalline rocks.

## 2. Basic Principles of FDEM

**Figure 1:**
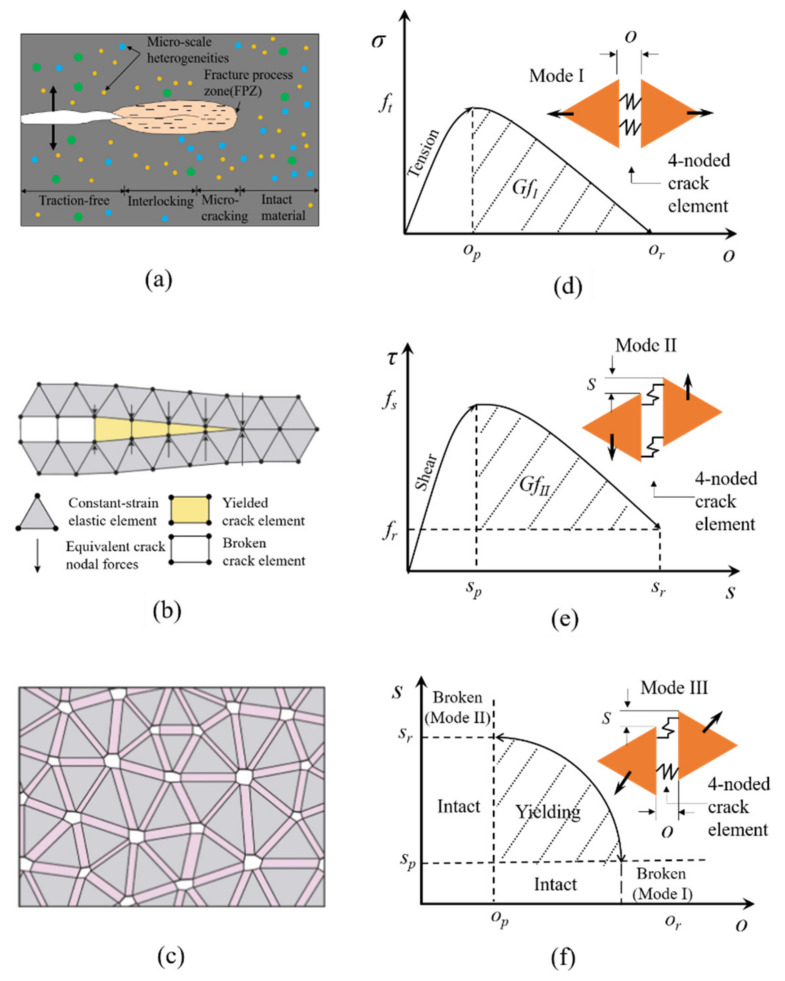
Failure models and failure criterion of CCEs in FDEM: (**a**) schematic of Fracture Process Zone (FPZ) in brittle geomaterials; (**b**) numerical representation of theoretical FPZ model in Irazu; (**c**) exaggerated view of 4-noded CCEs located along edges of all adjoining triangular finite elements; (**d**) tensile failure mode (mode I); (**e**) shear failure mode (mode II); and (**f**) mixed tension-shear failure criterion (mode III) (modified from Liu et al. [17]).

FDEM, proposed by Munjiza et al. [11], combines the advantages of the finite element method (FEM) and the discrete element method (DEM), continuous mechanical behaviors, such as the elastic deformation of brittle rocks, can be simulated by FEM, while discontinuous deformation behaviors such as damage and fracturing in brittle rocks can be simulated by the cohesive crack element (CCE) and the contact forces of blocks can be calculated by DEM. FDEM simulates the rock fracture process by introducing a model of the fracture process zone (FPZ) (Figure 1b) to simulate the initiation and propagation of microcracks, where the FPZ model is characterized by CCEs. Therefore, this method can capture the fracture evolution from continuity to discontinuity in brittle rocks, and any fracture trajectory can develop freely under the constraints of the mesh topology based on the stress and strain state.

This paper uses the Irazu 2D finite-discrete code based on FDEM [15]. In the Irazu 2D model, the domain is discretized with a topological mesh, which is composed of 3-noded constant-strain triangular elements and 4-noded CCEs. Each adjacent pair of triangles is connected with a CCE. Triangular elements represent rock blocks, and the elastic strain is simulated by triangular elements based on linear elastic continuum theory. The repulsive forces between contacting couples are calculated using a distributed contact force penalty function method, and the frictional forces between contacting couples are calculated using a Coulomb-type friction law. When the CCEs reach the critical condition of mode I (tensile fracture), mode II (shear fracture) or mixed mode III (tensile–shear fracture), the failure will occur. The connected triangular elements will be separated. After separation, they evolve into discontinuous blocks, and as the simulation progresses, the discrete blocks can undergo finite displacement and rotation, and new contacts can be created. The constitutive behavior and failure modes of CCEs are shown in Figure 1.

Because this method adopts several definite mechanical constitutive models for the initiation and coalescence of microcracks. Therefore, FDEM has been used to analyze and simulate the progressive fracturing process of brittle rock. More detailed principles of FDEM can be found in [15].

## 3. FDEM Simulation of Acoustic Emission

In FDEM, the strain energy generated from brittle rocks is simulated via the energy stored in triangular elements resulting from their elastic deformation. When the local CCEs reach the critical stress condition, then the strain energy stored in the triangular elements begins to release gradually via newly generated fractures. The released energy includes three parts: fracturing energy dissipated in CCEs during the yield stage, friction energy generated from the slipping of triangular elements, and kinetic energy of triangular elements which can be regarded as acoustic emission (AE) event energy. AE events can represent CCEs breakage. The energy of an AE event is equivalent to the maximum kinetic energy change of the CCE from entering yield state to complete failure.

**Figure 2:**
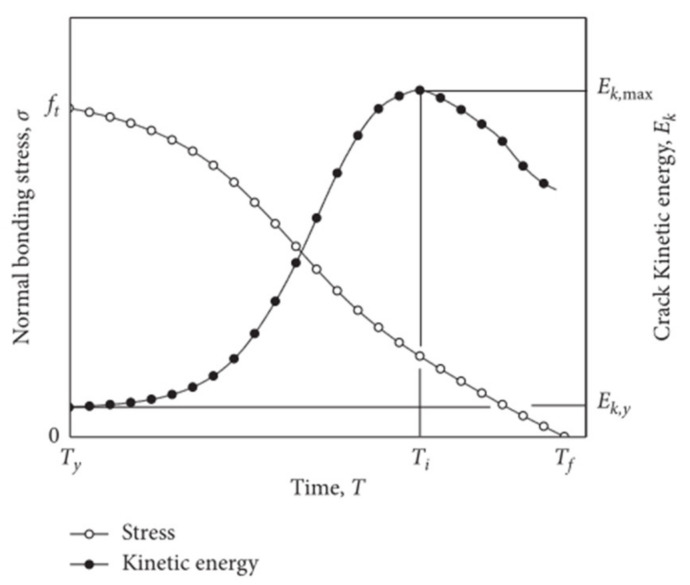
The evolution of normal bonding stress and kinetic energy (AE energy) of CCE nodes, $E_{k}$, as a function of time, *T*, for the tensile failing CCEs [17].

Since the brittle fracture in rocks occurs over a finite time interval, the initiation time is calculated by the change of the kinetic energy of the CCE. The evolution of the kinetic energy of the CCE nodes is correlated with the softening and rupture of a CCE (as shown in Figure 2). The initiation time,$\;T_{i}$, is assumed to be the time at which the kinetic energy of the CCE reaches a maximum.

Hazzard et al. proposed the algorithm of AE calculation for PFC model [18]. The detail calculation process in FDEM is as follows [19]:

(1) When the CCE reaches the peak strength, the kinetic energy of the CCE nodes is stored in memory as $E_{k,y}$:

(1) $$E_{k,y}=\sum_{i=1}^{4}m_{i}v_{i,y}^{2}$$

where $m_{i}$ and $v_{i}$, y are the nodal mass and nodal velocity at the time of yielding $T=T_{y}$, respectively.

(2) The kinetic energy, $E_{k}(t)$, of the CCE nodes is monitored until the CCE fails; the change in kinetic energy is calculated at each time step as:

(2) $$\Delta E_{k}(t)=E_{k}(t)-E_{k,y}$$

(3) The maximum change of $\Delta E_{k}$ in the whole process of CCE failure from the yielding moment $T_{y}$ to the failing moment $T_{f}$ can be regarded as the energy of AE event induced by each CCE breakage. It is calculated as follows and the initiation time of AE event corresponds to the moment $T_{i}$:

(3) $$E_{e}=\underset{\left[T_{y},T_{f}\right]}{\max}\Delta E_{k}(t)$$

(4) Finally, the event magnitude, $M_{e}$, can be calculated according to Gutenberg [20]:

(4) $$M_{e}=\frac{2}{3}(\mathrm{lg}E_{e}-4.8)$$

## 4. Numerical Grain-Based Model

**Figure 3:**
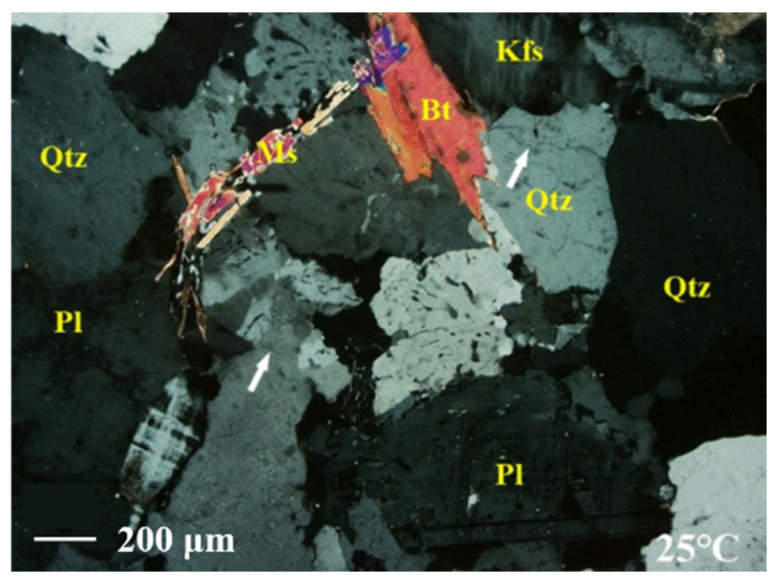
Micro-structure of Beishan granite (modified from [21] with permission from Elsevier).

**Figure 4:**
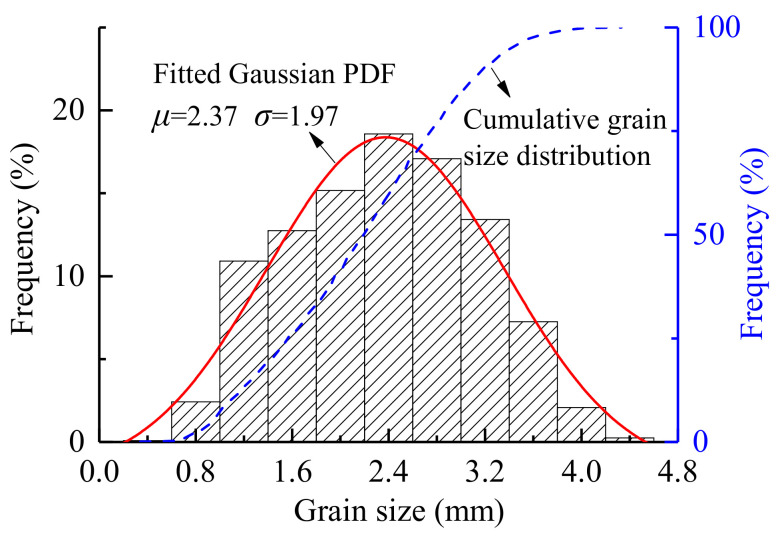
Grain size distribution of minerals in the grain-based model (PDF in the figure stands for the probability density function).

This paper establishes a heterogeneous numerical model of Beishan granite based on the results of experimental research [15]. Beishan granite is a fine-grained granite composed of quartz (Qtz), K-feldspar (Kfs), plagioclase (Pl), biotite (Bt) and muscovite (Ms), and the grain size ranges from 0.25 to 4.4 mm. The microstructure of Beishan granite mineral crystals is shown in Figure 3 [21]. This paper considers three mineral components, namely feldspar (Fsp), quartz (Qz) and mica (Ma), with mineral contents of 47%, 38% and 15%, respectively. The grain size of feldspar is largest, followed by quartz, and the size of mica is smallest. The grain sizes of minerals in the GBM are shown in Figure 4.

**Figure 5:**
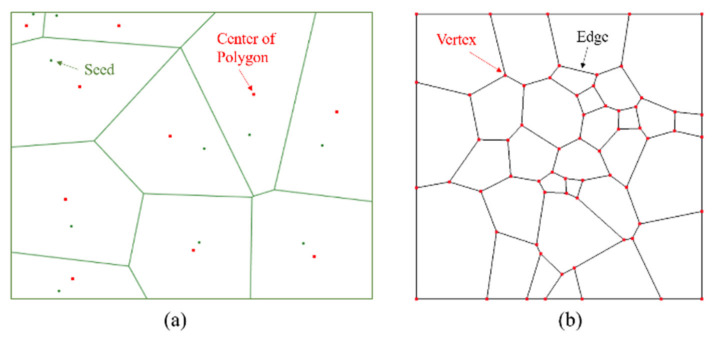
(**a**) typical Voronoi diagram. The red dots indicate the centers of the Voronoi polygons and the green dots indicate the seeds for generating Voronoi polygons; and (**b**) a combination of multiscale grains.

Neper [22], an open-source software package for 2D and 3D polycrystal generation and meshing, capable of generating multiscale tessellations, was utilized to generate the initial Voronoi region (Figure 5a).

**Figure 6:**
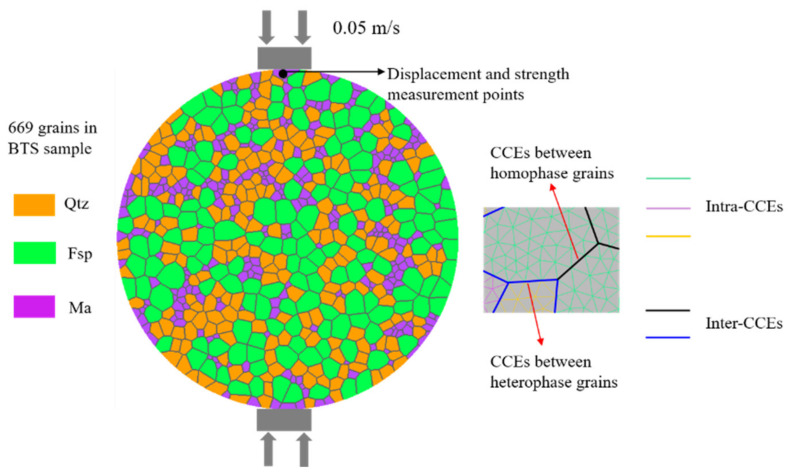
Schematic diagram of the grain-based model (GBM).

Based on the Poisson point process of Neper, Voronoi polygons were randomly generated locally (Figure 5b), and the radii of the equivalent area circles of the Voronoi polygons were set to obey a normal distribution, as shown in Figure 4. Simultaneously, the regularization process removes these small edges. Based on these Voronoi polygons, a meso-structure characterization geometric model with different mineral grain morphologies was constructed for Beishan granite, and the geometric model was imported into Gmsh [23] via a C++ subroutine, and then converted into a numerical model compatible with Irazu 2D. The model was discretized using an unstructured 2D Delaunay mesh comprising 30,619 triangular elements with an average element size of 0.5 mm. A mesh sensitivity analysis [24] exhibited that the element size represents an acceptable compromise between the computational demand and numerical accuracy. The GBM is illustrated in Figure 6.

## 5. Mesoscopic Parameter Verification of Grain-based Model

The size of the heterogeneous numerical sample constructed for Beishan granite is consistent with the laboratory sample size [16]. The sample is a disc with a diameter of 50 mm, and the upper and lower ends of the sample are equipped with loading platens. The two loading platens were simulated as moving towards the sample at a constant velocity of 0.05 m/s each. Although the simulated loading velocity is much greater than the experimental loading velocity in the laboratory, it has been demonstrated that a quasi-static condition is guaranteed at this loading velocity [25]. The numerical models were regarded as plane stress models, and a time step of 7 × 10^−7^ ms was adopted to ensure the numerical stability of the model in Irazu. A schematic diagram of the model under loading is presented in Figure 6. The force and displacement were obtained by monitoring the nodes of the upper loading platen.

The uniaxial tensile strength (*UTS*) can be calculated as follows

(5) $$UTS=P_{\max}/(\pi Rt)$$

where *P*_max_ is the maximum radial load, *R* is the radius of the disc and *t* is the thickness of the disc.

In the Irazu-GBM, the triangular elements inside the grains are connected by CCEs (shown as intra-CCEs in Figure 6), and the boundaries between adjacent grains are separated into heterophase boundaries (green lines in Figure 6) and homophase boundaries (black lines in Figure 6). Two types of boundaries are also simulated using CCEs (shown as inter-CCEs in Figure 6), but the strength parameters of the intra-CCEs are higher than those of homophase boundaries, and the strength parameters of homophase boundaries are higher than those of heterophase boundaries. This condition conforms to the objective law that the contact strength between mineral grains is lower than the strength of the mineral grains themselves, and can reflect the differences in the micromechanical properties of the boundaries between different grains. Therefore, this method can capture the spatial heterogeneity of the development of fracture section caused by the heterogeneity of mineral phases and grain sizes.

**Figure 7:**
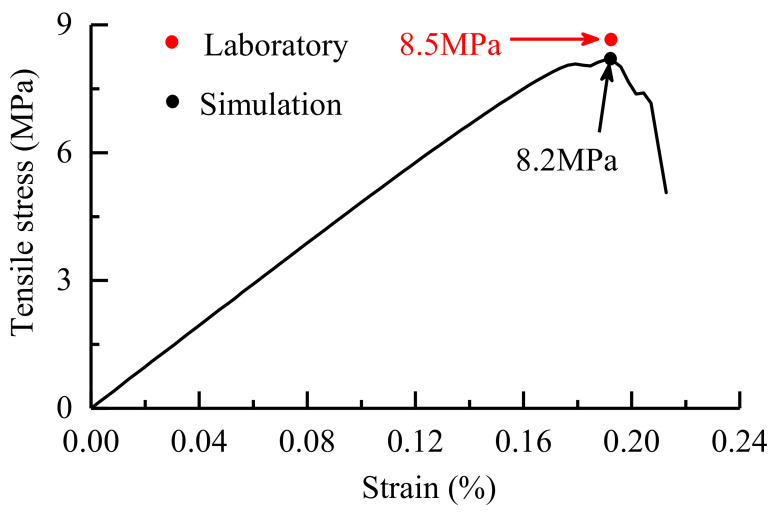
Tensile stress-strain curve of the calibration model.

**Figure 8:**
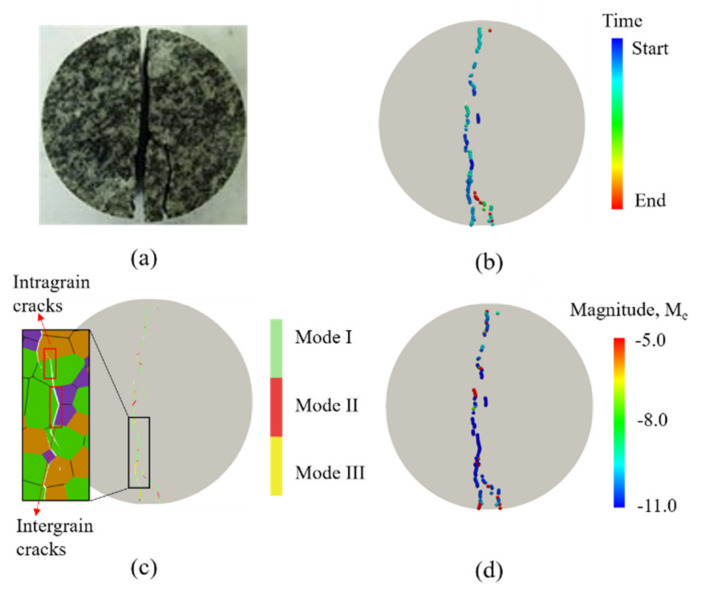
Comparison of the failure phenomenon: (**a**) fractured section of laboratory test (modified from [28] with permission from Elsevier); (**b**) acoustic emission (AE) evolution; (**c**) failure modes; and (**d**) magnitudes of AEs, M_e_.

The reference values [24,25,26] of the relevant physical and mechanical properties of common minerals in granite (Table 1) provide a reference basis for the calibration of the mesoscopic parameters of mineral grains of the synthetic Beishan granite samples. The mesoscopic parameters assigned to the intergrain boundaries (including both homophase and heterophase boundaries) of the model are obtained by an iterative calibration procedure [27] until the macroscopic strength parameters emerging from the numerical simulations closely match those obtained from laboratory testing. The mineral composition and the mesoscopic parameters of the mineral grains and contact boundaries of the synthetic Beishan granite samples are shown in Table 2. The UTS obtained by the numerical simulation is 8.2 MPa, as shown in Figure 7, with an error of 3.5% from the laboratory testing result [16]. Moreover, the fractured section obtained by the laboratory experiment [28] and the numerical simulation are in good agreement, as shown in Figure 8.

## 6. Analysis of the Effect of Meso-Heterogeneity on the Mechanical Properties

According to the meso-geometric heterogeneity of Beishan granite, this paper designs two numerical simulation schemes of Brazilian splitting tests to analyze the influence of meso-heterogeneity on the mechanical properties and failure laws from two perspectives, namely the grain size and grain orientation, as shown in Table 3.

### 6.1. Effect of the Grain Size

The grain size has a significant effect on the strength of brittle rock. The uniaxial compressive strength of rocks decreases with the increase of the grain size, which has been widely confirmed by laboratory experiments [7,29,30,31]. Specifically, Deng et al. [32] found that the peak load larger decreased as the grain size increased. Cowie et al. [33] analyzed laboratory experiments and found that the UTS of granite decreased with the increase of grain size. Nevertheless, in the conventional UDEC-GBM and PFC-GBM, the UCS and UTS of rock increase with the increase of grain size [34,35], which is contrary to these experimental findings. Therefore, the numerical analysis technology is in urgent need of improvement. Based on the PFC-GBM, Wong et al. [12] reduced the grain boundary strength to simulate the mechanism of the grain size effect. The FDEM-GBM has also been shown to be capable of simulating the grain size effect [13]. Hence, the effect of the grain size on the UTS and fracture evolution of granite can be studied in depth based on FDEM.

**Figure 9:**
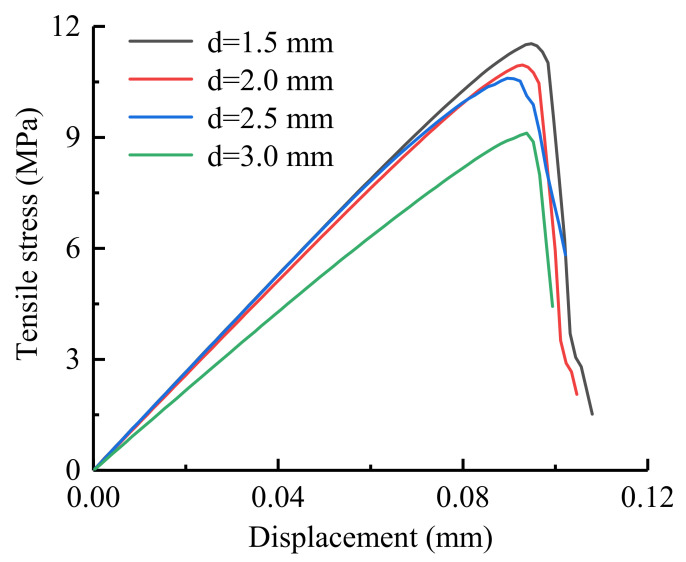
The tensile stress-displacement curves under different grain size conditions.

**Figure 10:**
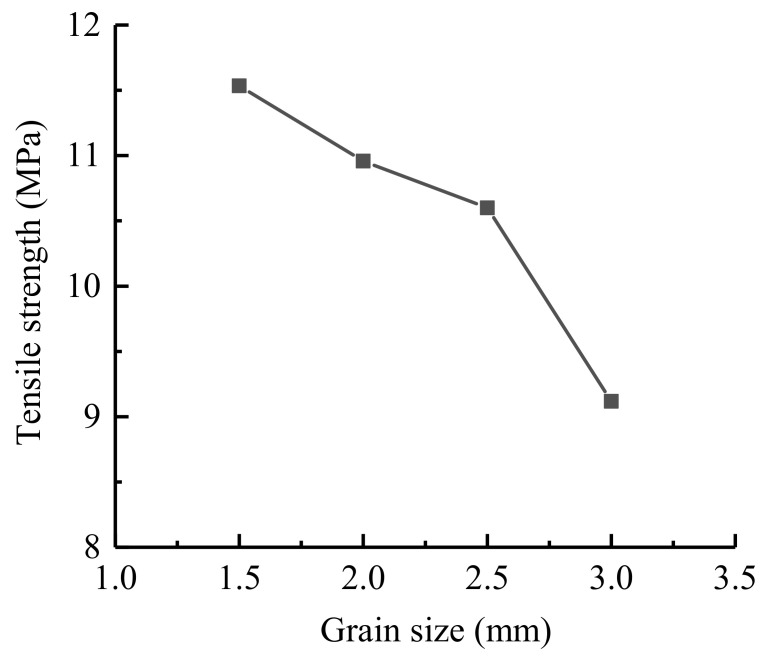
Relationship between the grain size and UTS.

In this chapter, Neper is used to generate four Brazilian disc models with different grain sizes (equivalent area circle diameters). The average grain sizes are approximately 1.5 mm, 2.0 mm, 2.5 mm and 3.0 mm, and the numbers of grains are 1016, 675, 418 and 301, respectively. The relationships between the tensile stress and displacement under different grain size conditions are shown in Figure 9, and the relationship between the grain size and UTS are shown in Figure 10, which shows that as the grain size increases, the UTS decreases, and the UTS ranges from 9.1 to 11.5 MPa.

**Figure 11:**
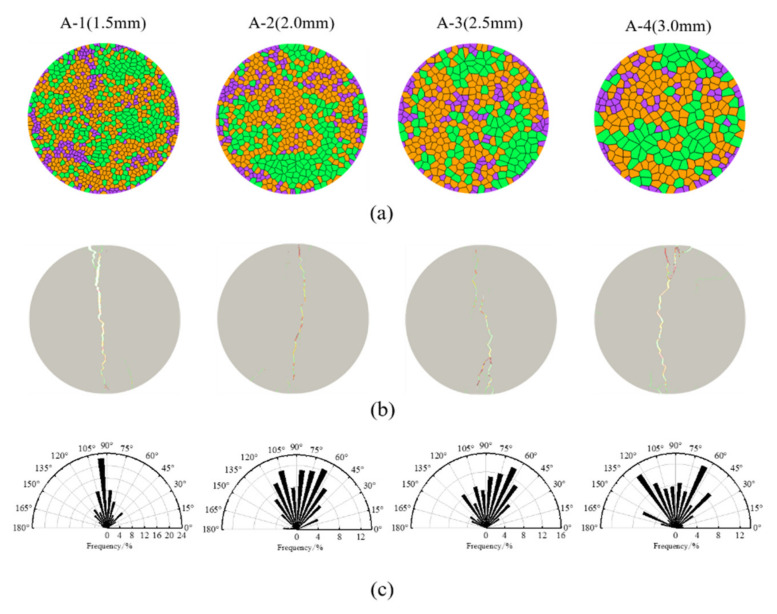
Characteristics of the samples and microcracks under different grain size conditions: (**a**) the four numerical samples; (**b**) the fracture paths (green, red and yellow segments represent tensile, shear and tensile-shear mixed cracks, respectively); and (**c**) the crack angle distributions.

The four numerical samples are shown in Figure 11a. The microcrack modes after the failure of the rock sample are shown in Figure 11b. The red, blue and yellow lines represent shear cracks, tensile cracks and mixed cracks, respectively, including intergranular and transgranular cracks. The statistical results of these microcracks are listed in Table 4. Most of the microcracks were intergranular tensile cracks, followed by transgranular mixed cracks. The total number of cracks and the number of transgranular cracks increased with the increase of the grain size, while the number of intergranular cracks decreased. This outcome indicates that if the grain size rises, more transgranular cracks will form. When the average grain size of the numerical model increased from 1.5 mm to 3.0 mm, the ratio of the number of intergranular tensile cracks to transgranular tensile cracks decreased from 29.8 to 6.8.

The paths of the microcracks after the failure of the four samples with different grain sizes are shown in Figure 11b. The cracks tended to pass through the intergrain boundaries, as the stiffness mismatch caused uncoordinated deformation. In addition, the main macroscopic fractures were formed by the expansion and coalescence of microcracks. With the existence of larger mineral grains, the shape of the main fracture changed from a straight line to a more complex multisegment curve or several curves. Therefore, the failure of heterogeneous rock includes the initiation, propagation and coalescence of microcracks, and microcracks are significantly controlled by the grain size.

The distributions of the crack inclination angles in the rock samples with different grain sizes are shown in Figure 11c. The crack inclination angle is defined as the angle between the microcrack direction and the horizontal direction measured positively anticlockwise. To plot Figure 11c, the inclination angle was divided into 12 groups with an interval of 15°. When the average grain size of the numerical model increased from 1.5 mm to 3.0 mm, the crack inclination angle changed from 75° ~ 105° to 30° ~ 165°, indicating that the grain size distribution has an important effect on the crack inclination angle. When the grain size decreased, the failure section became increasingly straight – the main reason for this phenomenon is that the heterogeneity of mineral grains will cause uncoordinated deformation, which will cause the tensile cracks to deviate from the center of the disc and produce off-center cracks. Therefore, the failure mode of granite is complicated.

**Figure 12:**
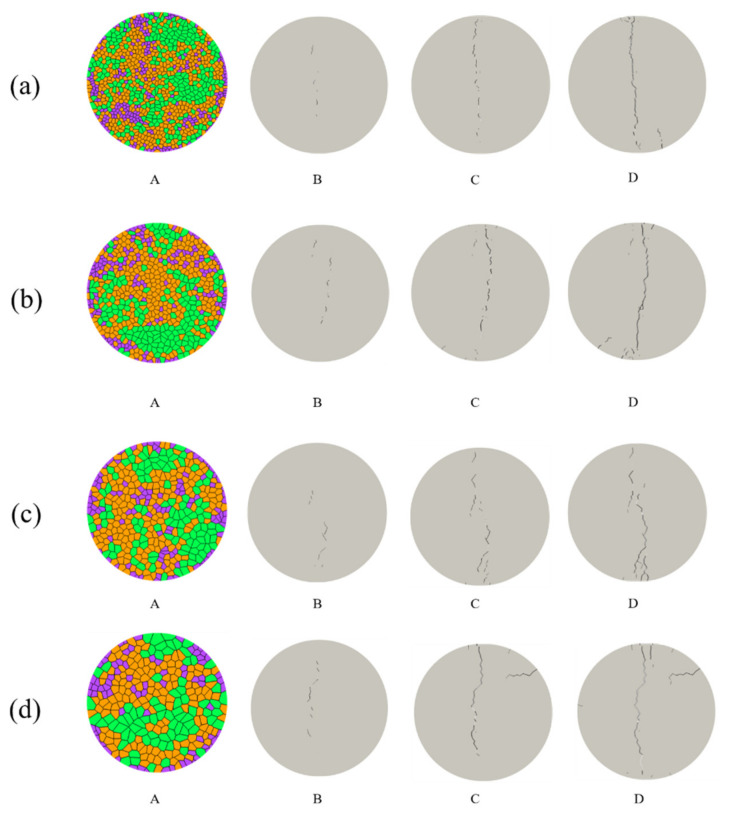
The variations of the fracture process under different grain size conditions: (**a**) size = 1.5 mm; (**b**) size = 2 mm; (**c**) size = 2.5 mm; (**d**) size = 3 mm (A represent the model; B, C and D represent the fracture process).

The variations of the fracture process under different grain size conditions are shown in Figure 12. The microcracks were firstly initiated in the center of the disc at the primary loading stage. Then, with increasing loading the tensile stress at the center of disk reaches the maximum value. Some visible macrocracks can be observed in the central region of disk, but not exactly the geometrical center. Reaching the peak, the main macro-fracture (brittle failure) is formed, which split the disk into two parts. Some sub-fractures are continuously formed. During the fracture developing process, orientation and shape of cracks are strongly influenced by the grain size. Most cracks are initiated at the boundaries between mineral grain develop mainly along weak paths characterized by contacts with low strength. We can observe intergranular cracks along boundary between mica and quartz grains and the transgranular cracks penetrating the mica grain.

In the numerical models, in terms of the mechanical properties of heterogeneous grain boundaries, the mechanical properties of grain boundaries between quartz and mica are the weakest, so the grain boundaries between quartz and mica are generally the zone where the intergranular cracks initiate. The mechanical properties of the heterogeneous grain boundaries are weaker than those of the homophase grain boundaries, and thus the heterogeneous grain boundaries are the main crack growth paths. The mechanical properties of intragain are relatively better than those of homophase and heterophase grain boundaries, so transgranular cracks do not easily occur. Simultaneously, the mechanical properties of mica are weaker than those of quartz and feldspar, so mica grains are the main zone where transgranular cracks occur.

**Figure 13:**
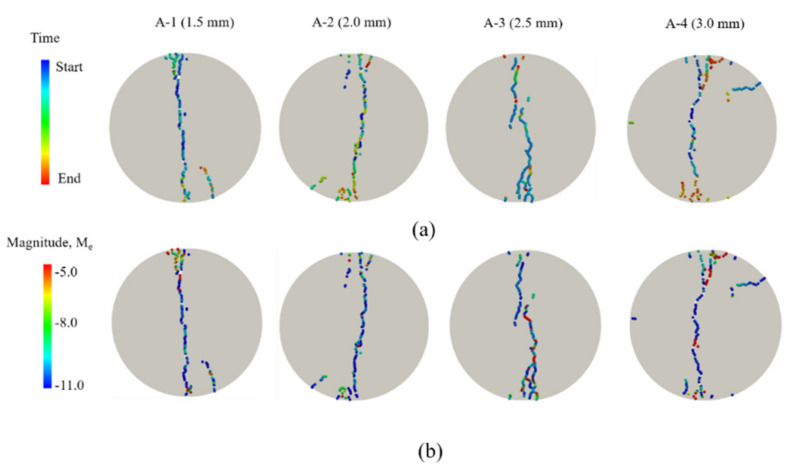
AE evolution and magnitudes of AEs under four different grain sizes conditions. (**a**) the AE evolution, (**b**) the magnitudes of AEs, M_e_, calculated from the kinetic energy of the sources using the technique illustrated in Section 3.

Figure 13a shows the AE evolution and Figure 13b the magnitudes of AEs under different grain sizes conditions corresponding to the different feature loading points. The AE events almost locate at the center of the disc in the former loading stage, and AE events are mainly at low-energy level. With rising axial stress, cracks gradually increase and locate discretely. Meanwhile, some discrete high-energy AE events occur in the rock model. During the whole process of splitting simulation, shear microcracks play the dominant role in energy release – particularly, they occur in later stage. Under different grain size conditions, the crack growth paths are significantly different. Finally, the oblique fracture belt rapidly extends and coalesces, and is characterized by the tensile failure mode. The larger the grain size is, the more complicated the crack propagation path and the greater the energy level differences. When the grain sizes are 1.5 and 2 mm, the fracture sections are smoother and straighter.

**Figure 14:**
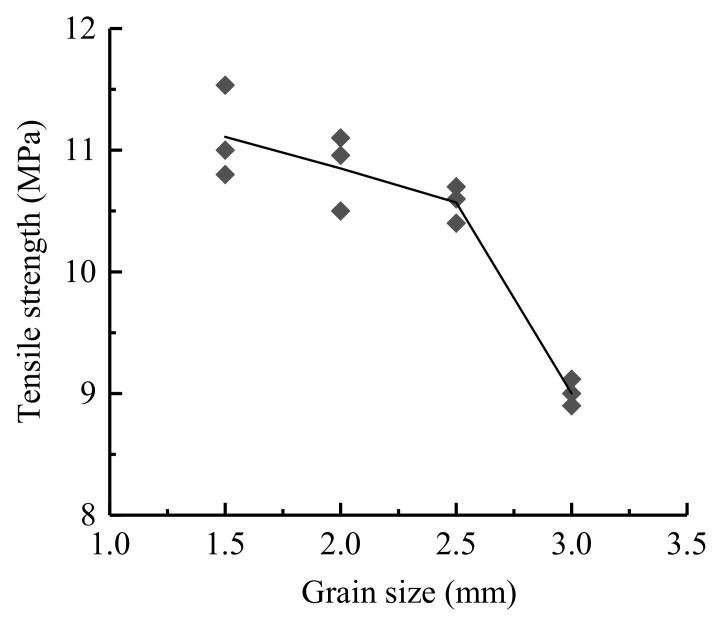
Relationship between the grain size and UTS.

In order to consider the influence of mineral distribution under each grain size condition, we generated three samples for each grain size. We obtain different tensile strengths when we choose different mineral distributions (as shown in Figure 14). We could find that with the increase of the grain size, the fluctuations of UTS were reduced. The possible reason for this is that there are fewer heterophase boundaries when the grain size is smaller.

### 6.2. Effect of the Preferred Grain Orientation

**Figure 15:**
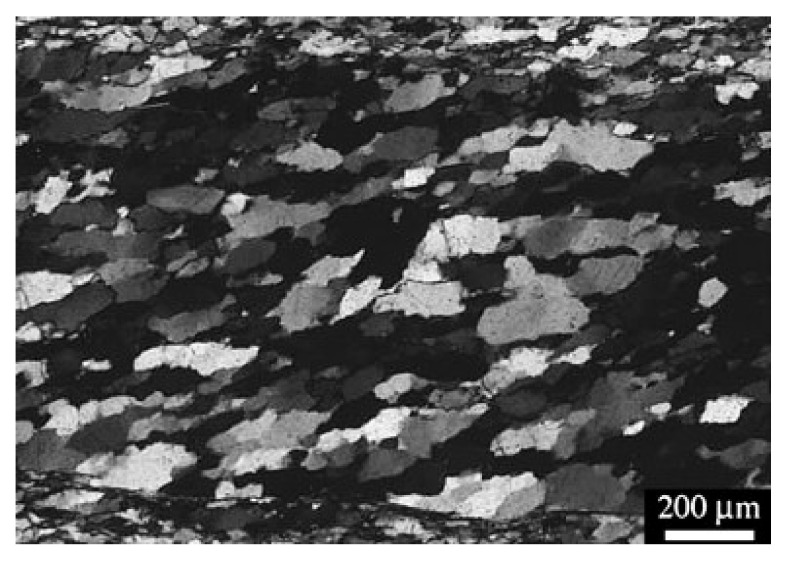
Microstructure of the quartz in granite [14] with permission from Elsevier.

Recrystallized quartz, as the primary mineral in granite, can exhibit obviously preferred orientation in some cases, as illustrated in Figure 15 [14]. However, existing GBMs cannot control the grain size distribution and preferred grain orientation at the same time. To resolve this problem, in this paper, the inverse Monte Carlo (IMC) algorithm is used to generate Voronoi polygons with specified grain size and grain orientation distribution characteristics.

The IMC algorithm assumes that the grain area follows a lognormal distribution, and the characteristics depend on the mean *μ*(*s*) and standard deviation *σ*(*s*). Given the area of the model and the number of grains, the grain size distribution depends only on the coefficient of variation *COV*(*s*), which is equal to the ratio of the standard deviation *σ*(*s*) to the mean *μ*(*s*). The smaller the *COV*(*s*) is, the more homogeneous the grain size distribution. At the same time, the angle between the maximum Feret diameter and the horizontal direction is defined as the grain orientation (*φ*), and its distribution takes the form of a normalized cosine function. More details regarding the IMC algorithm can be found in [36]. The formulas used to calculate the mean *μ*(*s*) and standard deviation *σ*(*s*) are

(6) $$\mu (s)=N_{P}/S_{D}$$

(7) $$\sigma (s)=COV(s)\cdot (N_{P}/S_{D})$$

where $N_{P}$ is the number of grains and $S_{D}$ is the area of the rock sample.

Using this method, a series of models with similar grain size distributions and different preferred orientations were generated, as shown in Table 5. The numerical models generated by the IMC method successfully present the expected statistical distributions of the grain size and preferred orientation. The grain size coefficient (*S*_o_) was used to measure the heterogeneity of the numerical model, where the higher the value of *S*_o_ is, the more heterogeneous the model. The models were generated in MATLAB and transferred to Gmsh via a C++ subroutine. The formula used to calculate the values of *S*_o_ [34] shown in the table is expressed as

(8) $$S_{o}=\sqrt{Q_{25\%}/Q_{75\%}}$$

where *Q*_25%_ and *Q*_75%_ correspond to the diameters smaller than 25% and 75%, respectively, of the grains on the grain size cumulative frequency diagram.

**Figure 16:**
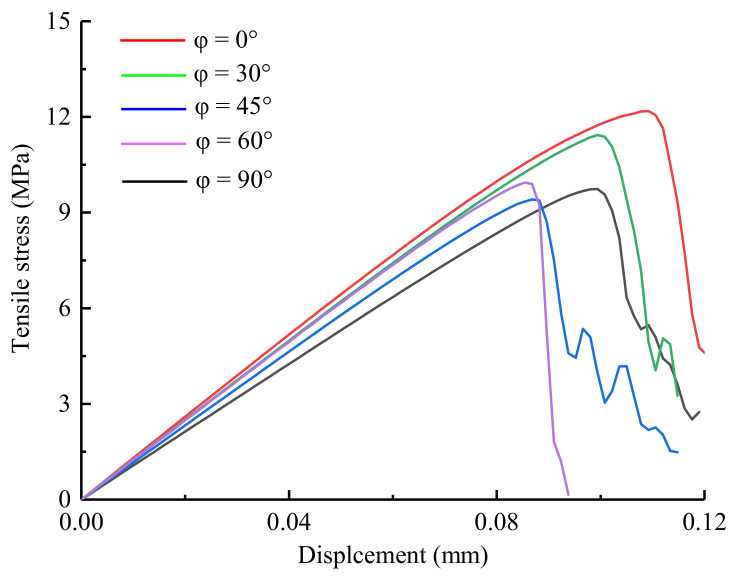
The tensile stress-displacement curves under different preferred grain orientation conditions.

**Figure 17:**
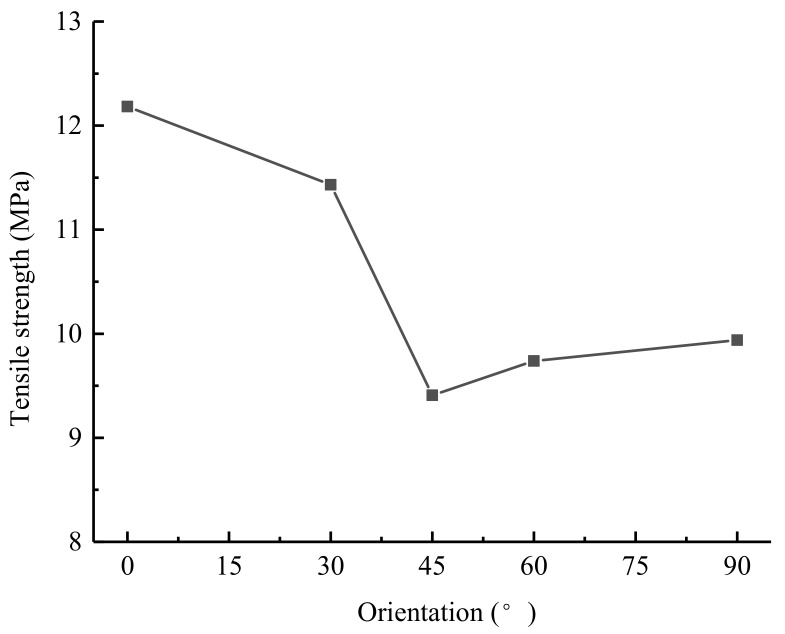
Relationship between the tensile strength and the grain orientation.

The tensile stress-displacement curves under different grain orientations are shown in Figure 16, and the relationship between the UTS and the grain orientation is shown in Figure 17. As the grain orientation increased, the UTS presented a “V-shaped” distribution characteristic; the UTS between 45° and 90° were lower than those between 0° and 30°, and the UTS were between 6.7 and 8.7 MPa. Indeed, Ghazvinian et al. [8] conducted an experiment on the anisotropic mechanical behavior of Cobourg limestone, and found that the strength and failure mode of the rock changed with the preferred grain orientation. Hence, as the UTS at 0° loading angle increased with the decrease of preferred grain orientation, the simulation results in this paper are basically consistent with the conclusions of previous experiments.

Tavallali et al. [37] proposed a method for calculating the mechanical energy applied by calculating the area under the force-displacement curve, which can be expressed as

(9) $$W=\frac{1}{2}P_{\max}\cdot \Delta S$$

where *W* is the mechanical energy exerted by the testing machine on the rock sample, *P*_max_ is the ultimate load during the loading process andΔS is the axial displacement corresponding to the ultimate load.

From an energy perspective, Beishan granite is extremely brittle; the stress linearly increases to the peak before falling, and it does not show an obvious yielding stage. Almost all the external energy absorbed by the rock is converted into elastic strain energy, which is stored inside the sample. Thus, the mechanical energy (*W*) input by the testing machine can be used to measure the energy storage capacity of the rock in the Brazilian splitting test. The grain orientation exerted a considerable influence on the energy storage capacity of granite. With the increase of grain orientation, the energy storage capacity decreased significantly. As shown in Figure 16, the greater the UTS is, the stronger the energy storage capacity. As revealed by the Brazilian splitting test, the energy storage capacity of the granite was negatively correlated with the grain orientation. From the perspective of engineering rock breaking, understanding the energy storage capacity of rocks and improving energy utilization are of great significance for maintaining the integrity of the rock masses.

**Figure 18:**
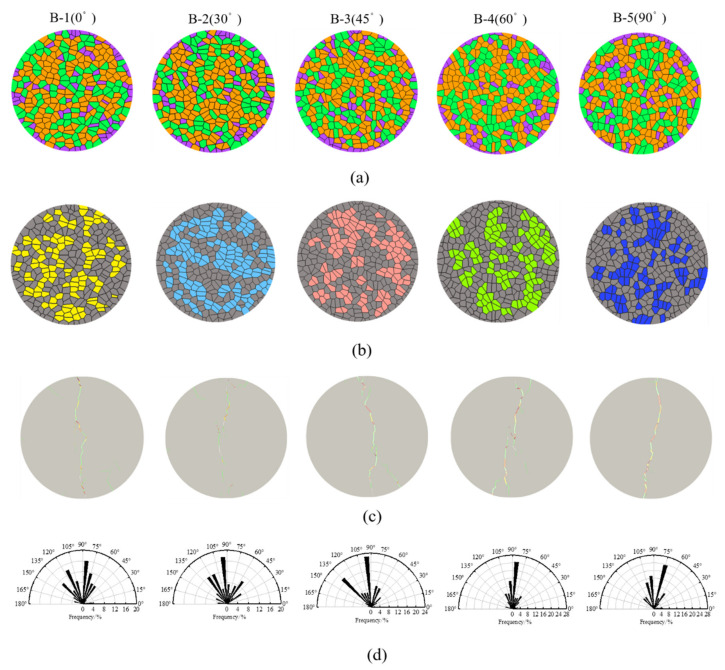
Characteristics of the sample and crack with five different preferred grain orientations: (**a**) the numerical samples; (**b**) the models with the preferred grain orientations (highlight grains represent grains with preferred orientations); (**c**) the fracture paths (green red and yellow segments represent tensile, shear and tensile-shear mixed cracks, respectively); and (**d**) the crack angle distributions.

The numerical samples are shown in Figure 18a, and the grains with the preferred orientations are shown in Figure 18b. Figure 18c shows the crack paths and crack failure modes under different grain orientation conditions. The red, blue and yellow lines represent shear cracks, tensile cracks and tensile-shear mixed cracks, including intergranular and transgranular cracks. The shapes of the fracture sections for the five different grain orientation models are quite different. Nevertheless, the preferred grain orientation provides a preferential path for the growth of microcracks, and thus the failure mode of the rock changes with the preferred grain orientation. Tensile cracks are dominant among the samples with preferred orientations of 0°, 30° and 45°, while for samples with 60° and 90°, tensile-shear mixed cracks are observed to increase.

Figure 18d shows the distributions of the crack inclination angles under different grain orientation conditions. When the grain orientation is 0°, the crack inclination angles are distributed mainly between 45° and 135°; when the grain orientation is 30°, the crack inclination angles are distributed mainly between 90° and 130°; when the grain orientation is 45°, the crack inclination angles are distributed mainly between 90° and 100°, and between 130° and 140°; when the grain orientation is 60°, the crack inclination angles are mainly distributed between 50° and 120°; and finally, when the grain orientation is 90°, the crack inclination angles are distributed mainly between 70° and 110°. The preferred grain orientation clearly has a significant impact on crack propagation. The reason for this is that the damage of the sample is divided into two main processes: the sample is firstly damaged locally along the grain boundaries parallel to the loading direction, and the grain boundaries are always in weak contact; then, these local cracks provide a dominant direction for further damage, which propagates from the crack tip before expanding and penetrating the sample. Finally, these cracks coalescence and cause the sample to fail.

**Figure 19:**
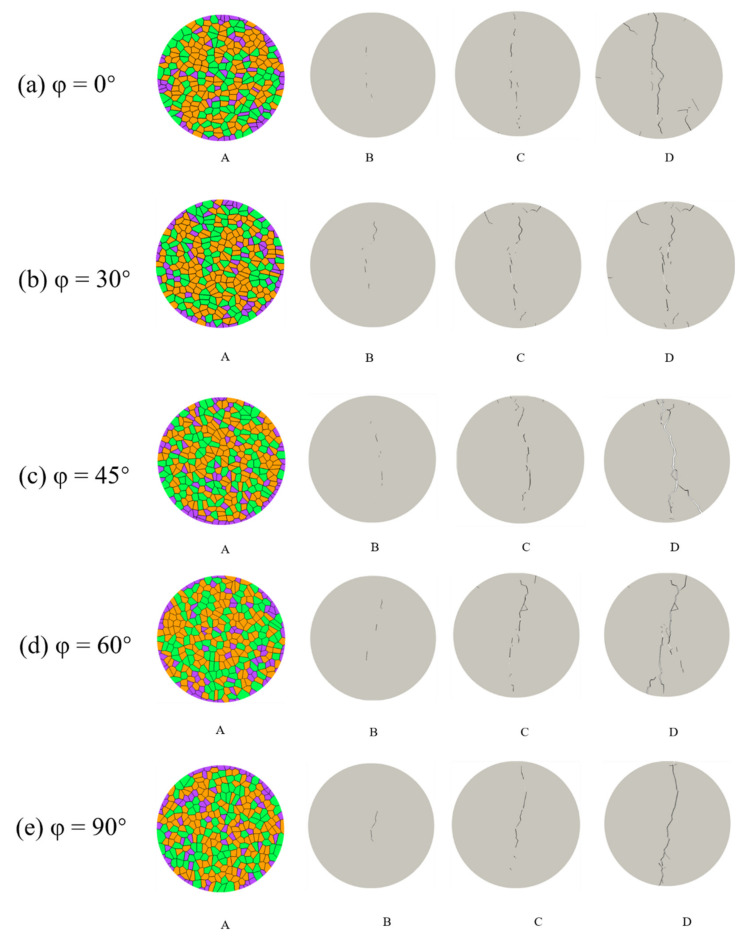
The variation of the fracture process under different preferred grain orientation conditions: (**a**) orientation = 0°; (**b**) orientation = 30°; (**c**) orientation = 45°; (**d**) orientation = 60°; and (**e**) orientation = 90° (A represents the model; B, C and D represent the fracture process).

The variations of the fracture process under different preferred grain orientation conditions are shown in Figure 19. During the fracture developing process, orientation and shape of cracks are strongly influenced by the grain orientation. Most cracks are initiated at the boundaries between mineral grain, developing mainly along weak paths characterized by contacts with low strength, and the fracture paths become more complicated during the later stage of loading.

**Figure 20:**
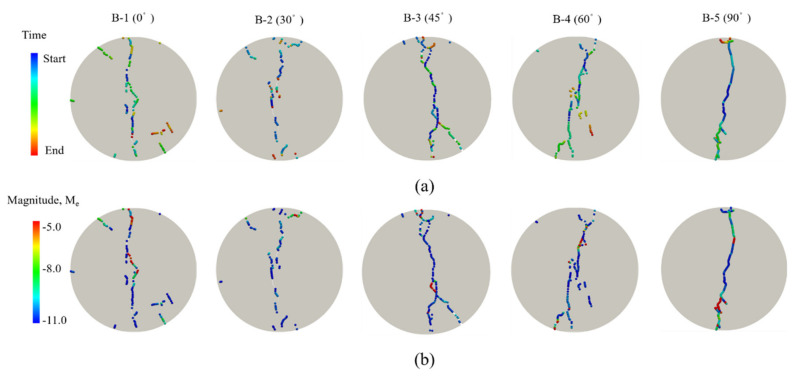
AE evolution and magnitudes of AEs under different grain orientation conditions: (**a**) the AE evolution; and (**b**) the magnitudes of AEs, M_e_, calculated from the kinetic energy of the sources using the technique illustrated in Section 3.

Figure 20a shows the AE evolution and Figure 20b shows the magnitudes of AEs under different grain orientation conditions corresponding to the different feature loading points. The AE events almost locate at the center of the disc in the former loading stage, and AE events are mainly at low-energy level. With rising axial stress, cracks gradually increase and locate discretely. Meanwhile, some discrete high-energy AE events occur in the rock model. During the whole process of splitting simulation, shear microcracks play the dominant role in energy release – particularly, they occur in the later stage. Under different preferred grain orientation conditions, the crack growth paths are significantly different. Finally, the oblique fracture belt rapidly extends and coalesces, and is characterized by the tensile failure mode. When the preferred orientations are 30°, 45° and 60°, the fracture sections are more complicated.

## 7. Conclusions

On the basis of the Voronoi grain-based modeling method, this paper reproduced the continuous-discontinuous fracture process of heterogeneous rock under the framework of the combined finite-discrete element method (FDEM). Considering the mechanical strength and elastic deformation properties of Beishan granite, the grain-based model (GBM) was introduced into the meso-mechanical analysis of granite samples and a series of numerical Brazilian splitting numerical tests were carried out to analyze the effect of the grain size and preferred orientation. The main results are summarized as follows:

1. The FDEM-GBM considers the complexity of the contacts between different mineral grains in the rock and the physical and mechanical properties of different mineral phases; therefore, the FDEM-GBM has the ability to simulate the effect of the grain size in heterogeneous rocks.
2. Because the mineral grain has significant influence on the crack propagation paths, the FDEM-GBM can capture the location of fractures more accurately than the conventional models. Shear cracks occur near the loading area, while tensile and tensile-shear mixed cracks occur far from the loading area. The applied stress must overcome the tensile strength of the intergrain contact.
3. The UTS and the ratio of the number of intergrain tensile cracks to the number of intragrain tensile cracks are negatively correlated with the grain size. During the whole process of splitting simulation, shear microcracks play the dominant role in energy release; particularly, they occur in later stage. Under different grain sizes conditions, the crack growth paths are significantly different. The larger the grain size is, the more complicated the crack propagation path. When the grain sizes are 1.5 and 2 mm, the fracture sections are smoother and straighter.
4. With the increase of preferred grain orientation, the UTS presents a “V-shaped” characteristic distribution. During the whole process of splitting simulation, shear microcracks play the dominant role in energy release; particularly, they occur in later stage. Under different preferred grain orientation conditions, the crack growth paths are significantly different, especially 30°, 45° and 60°. When the preferred orientations are 30°, 45° and 60°, the fracture sections are more complicated.
5. The preferred grain orientation considerably influences the energy storage capacity of Beishan granite. With the increase of grain orientation, the energy storage capacity decreases significantly. The greater the UTS is, the higher the energy storage capacity.

Since the mesoscopic simulations of heterogeneous rock conducted herein are implemented under the framework of the combined finite-discrete element method (FDEM), the model has many mesoscopic parameters, which require much “trial and error” work. Consequently, further work is needed to reduce the difficulty of encountering too many mesoscopic parameters in the calibration process.

## Figures and Tables

**Table 1:** Physical and mechanical parameters of the primary minerals in granite [24,25,26].

Mineral	Density (kg·m^−3^)	Tensile Strength (MPa)	Young’s Modulus (GPa)	Poisson’s Ratio (-)
K-feldspar	2560	5–10	69.8	0.28
Plagioclase	2630	5–10	88.1	0.26
Quartz	2650	10–11	94.5	0.08
Biotite	3050	4–7	33.8	0.36

**Table 2:** Mesoscopic parameters of the grain-based model in Irazu 2D.

	Property	Qz	Fsp	Ma
Intragrain	Density (kg·m^−3^)	2600	2600	3050
	Young’s modulus (GPa)	80	70	40
	Poisson’s ratio (-)	0.07	0.26	0.27
	Friction coefficient (-)	1.2	1.2	1.2
	Cohesion (MPa)	25	25	25
	Tensile strength (MPa)	20	15	10
	Mode I fracture energy (J·m^−2^)	900	300	600
	Mode II fracture energy (J·m^−2^)	1800	600	1200
	Fracture penalty (GPa)	400	350	200
	Normal penalty (GPa·m)	80	70	40
	Tangential penalty (GPa·m^−1^)	800	700	400
		Qz-Qz	Fsp-Fsp	Ma-Ma
Homophase boundary	Friction coefficient (-)	1.1	1.1	1.1
	Cohesion (MPa)	20	20	20
	Tensile strength (MPa)	15	10	10
	Mode I fracture energy (J·m^−2^)	700	250	450
	Mode II fracture energy (J·m^−2^)	1400	500	900
	Fracture penalty (GPa)	200	175	100
	Normal penalty (GPa·m)	40	35	20
	Tangential penalty (GPa·m^−1^)	400	350	200
		Qz-Fsp	Qz-Ma	Fsp-Ma
Heterophase boundaries	Friction coefficient (-)	0.9	0.9	0.9
	Cohesion (MPa)	20	20	20
	Tensile strength (MPa)	10	6	6
	Mode I fracture energy (J·m^−2^)	50	20	20
	Mode II fracture energy (J·m^−2^)	500	200	200
	Fracture penalty (GPa)	350	200	275
	Normal penalty (GPa·m)	70	40	55
	Tangential penalty (GPa·m^−1^)	700	400	550

**Table 3:** Numerical test schemes.

Scheme	Grain Size (mm)	Grain Orientation (º)
1	1.5, 2.0, 2.5, 3.0	-
2	-	0, 30, 45, 60, 90

**Table 4:** Summary of microcrack types after Brazilian splitting tests.

Sample	Number of Different Types of Microcracks					
	Intergranular Cracks			Transgranular Cracks		
	Mode I	Mode III	Mode II	Mode I	Mode III	Mode II
A-1	119	23	2	4	68	1
A-2	121	43	2	7	48	3
A-3	150	56	3	10	50	6
A-4	130	70	3	19	52	10

**Table 5:** Characteristics of the numerical samples with different preferred grain orientations.

Sample	Grain Number, *N*_P_	Grain Size Coefficient, *S*_o_	Preferred Orientation, *φ* (°)
B-1	303	1.06	0
B-2	319	1.07	30
B-3	329	1.06	45
B-4	328	1.07	60
B-5	321	1.06	90

## Data Availability

Data sharing is not applicable to this article.
